# Predictors of exclusive breastfeeding duration among 6–12 month aged children in gurage zone, South Ethiopia: a survival analysis

**DOI:** 10.1186/s13006-017-0107-z

**Published:** 2017-04-21

**Authors:** Abebaw Wasie Kasahun, Wako Golicha Wako, Meron Worku Gebere, Gebremariam Hailemichael Neima

**Affiliations:** Department of Public Health, College of Medicine and Health Sciences, Wolkite University, Wolkite, Ethiopia

**Keywords:** Exclusive breastfeeding, Predictors, Survival analysis, South Ethiopia

## Abstract

**Background:**

Exclusive breastfeeding is giving only breast milk to an infant from birth up to six months of age, with the exception of medications and vitamins. For the first six months of life, breast milk alone is the ideal nourishment to meet the nutritional demand of the growing child. Although breastfeeding is a universal practice, in Ethiopia only 52% of children aged less than six months old were exclusively breastfed. The study aimed to investigate the predictors of exclusive breastfeeding duration among women who had children aged between 6–12 months in Gurage zone, South Ethiopia.

**Methods:**

A mixed method cross-sectional study was conducted to assess predictors of exclusive breastfeeding duration in south Ethiopia. Eight hundred and twenty eight study participants were recruited using a multistage sampling technique for the quantitative survey. Interviewer administered close ended questionnaire was used to collect the quantitative data. Data were entered using Epi Data and analyzed using SPSS version 21. The Kaplan-Meier curve with log rank test was used to compare the survival difference due to the selected covariates. A binary and multivariable Cox regression model was used to identify the independent predictors of exclusive breastfeeding duration. Three focus group discussions were conducted to generate the qualitative data. Qualitative data is transcribed and analyzed by thematic approach using open-code software.

**Results:**

The median duration of exclusive breastfeeding was six months. About 21.9% of women introduced complementary food before six months of child age. Women with education status of diploma and above (Adjusted Hazard Ratio [AHR]: 2.89, 95% CI: 1.05, 7.97), perceived inadequate breast milk (AHR: 11, 95% CI: 6.7, 18.0) and cesarean section delivery (AHR: 3.8, 95% CI: 2.0, 7.2) were more likely to cease exclusive breastfeeding before six months of child age; while women who had infant feeding counseling during postnatal care (AHR: 5.1, 95% CI: 2.5, 10.23) were less likely to cease exclusive breastfeeding before the child was six months of age.

**Conclusions:**

A significant proportion of women cease exclusive breastfeeding before the recommended six months duration. Maternal education of diploma and above, perceived inadequacy of breast milk, cesarean section delivery, postnatal counseling on child feeding are factors significantly associated with the duration of exclusive breastfeeding. Encouraging behavioral change and improving communication regarding the duration of exclusive breastfeeding, and increasing the utilization of postnatal counseling about exclusive breastfeeding are recommended.

## Background

Exclusive breastfeeding (EBF) is defined as giving only breast milk to an infant from birth up to six months of age without giving any other food items including water, except for medicine and vitamins. For the first six months of life, breast milk alone is the ideal nourishment for infants, meaning that any other food is not needed [1].

Breast milk promotes sensory and cognitive development, and protects the infant against infectious and chronic diseases. Exclusive breastfeeding reduces infant mortality and aids a quicker recovery during an illness. It is also important for mothers as it may delay the return of fertility. Lack of exclusive breastfeeding during the first six months of life is the most important risk factor for infant and childhood morbidity and mortality, including the life-long impacts of poor school performance, reduced productivity, and impaired intellectual development [2–4]. Early introduction of complementary foods increases infant morbidity and mortality, by reducing the ingestion of protective factors present in breast milk and increasing the exposure to sources of contamination [5].

Suboptimal breastfeeding is the third commonest risk factor to disability adjusted life years in Ethiopia [3]. The Ethiopian government recommends exclusive breastfeeding for the first six months of age, however only 52% of under-five year old children were exclusively breastfed at the time of the survey. This figure declined to 32% for children 4–5 months old [6].

There is limited evidence to inform policy makers of the factors affecting exclusive breastfeeding duration in Ethiopia. Therefore this study aimed to investigate the predictors of exclusive breastfeeding duration in Gurage zone, South Ethiopia.

## Methods

### Study design and setting

A quantitative cross-sectional study design complemented with a qualitative method was conducted to assess predictors of exclusive breastfeeding duration among women who had 6–12 month old children in Gurage zone, South Ethiopia, from December 2015 to April 2016. The center of Gurage zone is located 154 km from Addis Ababa, the capital of Ethiopia. The study area are rural districts with a small town at the center for each district.

### Population and sample size

Women who have 6–12 months old children and who initiated breastfeeding in the selected districts of Gurage zone were the source population.

The sample size was calculated using two population proportion formula, using Epi Info7 software considering the following assumptions: 95% confidence level, 80% power and the proportion of exclusive breastfeeding duration to six months among women who have not received antenatal care is 27.7% with Adjusted Odds Ratio (AOR) 1.7 as compared to women who have received antenatal care [7]. With the design effect of 1.5 and allowing for a 5% non-response rate, the required sample size was 850 mother-child pairs.

### Sampling procedure

Multi-stage sampling technique was used to recruit study subjects. First, three districts were randomly selected from a total of 13 districts. Second, two rural and one urban kebele (smallest administrative unit) were selected from each district after stratifying a kebele as urban and rural. The sample size was proportionally allocated to each kebele depending on population size. Finally, the study participants were selected by simple random sampling using family register at each kebele as a frame.

### Data collection

Quantitative data were collected by the interviewer who administered the pretested structured questionnaire. The questionnaire included sociodemographic, women’s reproductive health, breastfeeding attitude and breastfeeding practice components. The questionnaire was developed in English and translated to Amharic by fluent speakers of both languages and; then translated back to English by different language experts to ensure language consistency. The Amharic version questionnaire was used to collect data. Quantitative data were collected by diploma holder nurses and health extension workers who are fluent speakers of local languages.

In addition to quantitative data, three focus group discussions were conducted, one in an urban and two in rural settings. Each focus group discussion consisted of eight participants who were not involved in the quantitative survey. Focus group participants had similar sociodemographic characteristics with the quantitative survey participants. Women who were currently breastfeeding 6–12 month old children were purposively selected for focus group discussions. Semi-structured question guide was used to facilitate the discussions. The question guide was developed by reviewing relevant literatures to area of interest. Appropriate questioning was done by the investigators. Focus group discussions were audio recorded and note taking was done. The audio was transcribed immediately after the discussions.

### Data measures

Exclusive breastfeeding duration was assessed using a ‘since birth’ recall approach. Mothers were asked “what was the age of your child (“Name”) when you first gave food items other than your breast milk including water.” Responses were recorded in months. The status of exclusive breastfeeding was determined based on the reported duration of exclusive breastfeeding. Women who had reported exclusive breastfeeding below six months were considered as events and those who exclusively breastfeed to six months and beyond were censored.

Maternal attitude towards breastfeeding was assessed using the Iowa Infant Feeding Attitude Scale. The scale uses the five point Likert scale range of strongly agree (5) to strongly disagree (1) for positively worded statements and vice versa for negatively worded statements [8].

Education status was recorded based on respondents schooling status: Illiterate refers to women who cannot read and write. Can read and write only refers to women with no formal school attendance but who can read and write. Primary first cycle refers to women who attend only a class of grade 1^st^ - 4^th^, primary second cycle refers to women who completed first cycle and attend a class of grade 5^th^ - 8^th^. Secondary school refers to women who attends a class of grade 10^th^ - 12^th^ and diploma and above refers to women who graduate at college or university level course [9].

### Data analysis

Quantitative data were entered using Epi Data 3.1 and exported to SPSS version 21 for analysis. Principal component analysis was computed to rate household wealth using fixed and other household items for each participant. Five components were retained based on Eigen value greater than one and wealth index was ranked into five quintiles. Proportionality hazard assumption test was checked using log (−log) versus log (time) graph and time dependent Cox model for relevant variables. In both tests proportionality hazard assumption is satisfied.

Descriptive statistics like frequencies, proportions, mean and median were used to explain important variables in relation to the outcome variable. The Kaplan-Meier survival curve with the log rank test was used to compare survival of exclusive breastfeeding using selected variables. The effect of each variable on duration of exclusive breastfeeding is assessed using bivariate Cox regression model. Variables with *p* - value < 0.2 in bivariate Cox regression were further analyzed using multivariable Cox regression model. Adjusted Hazard Ratio (AHR) with 95% Confidence Interval (CI) and *p* < 0.05 was used to declare significance level of variables.

Qualitative data were transcribed, translated and synthesized by investigators. The content is exported into open code software and analyzed using thematic approach.

## Results

Eight hundred twenty eight respondents (828) were successfully interviewed; a response rate of 97.4%. About 50 responses were excluded from the analysis as a result major incompleteness and the final analysis was done using 778 respondents.

### Sociodemographic characteristics of respondents

Four hundred forty six (57.3%) respondents were rural residents and the rest were urban residents. Other sociodemographic characteristics of respondents are summarized in Table 1.

**Table 1:** Sociodemographic characteristics of women who had 6–12 months old child in Gurage zone, South Ethiopia, 2016 (*n* = 778)

Variables	Category	Frequency (*n)*	Percentage (%)
Child sex	Male	373	47.9
	Female	405	52.1
Maternal age (years)	15-19	15	1.9
	20-24	74	9.5
	25-29	289	37.1
	30-34	232	29.8
	35-39	150	19.3
	40-44	17	2.2
	45-49	1	0.1
Maternal religion	Orthodox Christian	419	53.9
	Islam	268	34.4
	Protestant	83	10.7
	Others	8	1.03
Area of residence	Urban	332	42.7
	Rural	446	57.3
Maternal marital status	Single	47	6.0
	Married	698	89.7
	Widowed	9	1.2
	Divorced	24	3.1
Maternal occupation	Housewife	541	69.5
	Farmer	62	8.0
	Merchant	104	13.4
	Daily laborer	22	2.8
	Employed	46	5.9
	Others	3	0.4
Maternal educational status	Illiterate	291	37.4
	Can read and write	178	22.9
	Primary –first cycle	99	12.7
	Primary – second cycle	98	12.6
	Secondary school	74	9.5
	Diploma and above	38	4.9

### Breastfeeding knowledge and practice

Seven hundred twenty six (93.3%) respondents have heard about exclusive breastfeeding duration while the rest 52 (6.7%) have no information regarding exclusive breastfeeding duration. Five hundred ninety three (76.2%) respondents mentioned the correct duration of exclusive breastfeeding and regarded as having good knowledge while the rest had poor knowledge. Three quarter of respondents (75.6%) initiated breastfeeding within the first hour after birth and 23.3% of respondents initiated breastfeeding within 24 h after birth while the rest few (1%) respondents initiated breastfeeding after 24 h of birth.

The mean and median duration of exclusive breastfeeding was 5.7 (95% CI: 5.6, 5.8) and 6 months respectively. The minimum and maximum duration of exclusive breastfeeding was one month and nine months respectively. The cumulative proportion of survival probability up to six months on exclusive breastfeeding was 78.1% as illustrated in the life Table 2.

**Table 2:** Life table for exclusive breastfeeding duration to the first six months of child age among women who had 6–12 months old child in Gurage zone, South Ethiopia, 2016

Time(months)	Number of surviving children	Proportion of surviving cases (%)	Event	Proportion of events (%)	Cumulative events	Remaining cases
0	778	100	0	0	0	778
1	778	100	7	0.9	7	771
2	771	99.1	1	1	8	770
3	770	99	26	4.4	34	744
4	744	95.6	62	12	96	682
5	682	88	74	21.9	170	608
6	609	78	(censored)	21.9	170	608

### Factors affecting duration of exclusive breastfeeding

**Fig. 1:**
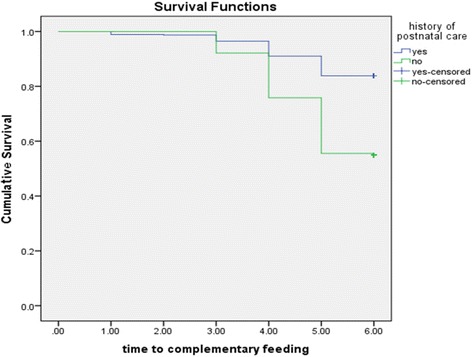
Cumulative Survival probability of exclusive breastfeeding practice for women who had and had not postnatal care, South Ethiopia, 2016. (log rank test <0.05)

The cumulative survival probability of exclusive breastfeeding to six months was significantly higher for women who had postnatal care (PNC) as compared to women who had no PNC visit (*log rank test, p < 0.001*) as shown in Fig. 1.

**Fig. 2:**
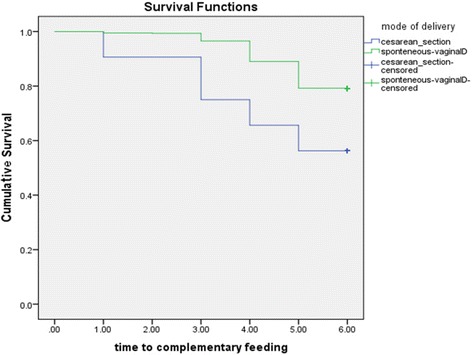
Cumulative Survival probability of exclusive breastfeeding practice for women who gave birth spontaneously and through cesarean section, South Ethiopia, 2016. *(log rank test <0.05)*

A child’s cumulative survival probability of exclusive breastfeeding up to six months of age was significantly different between women who had a spontaneous vaginal birth, and women who gave birth by cesarean section (*log rank test, p < 0.05)*. Women who gave birth spontaneously were more likely to sustain exclusive breastfeeding to six months as compared to women who gave birth through cesarean section as shown in Fig. 2.

**Fig. 3:**
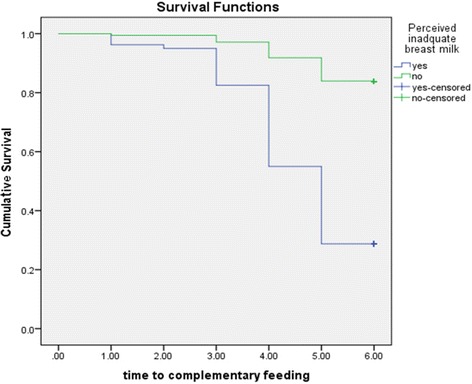
Cumulative Survival probability of exclusive breastfeeding practice in relation to women’s perception on adequacy of their breast milk, South Ethiopia, 2016. (log rank test < 0.050

Women’s perceived inadequacy of breast milk significantly influences duration of exclusive breastfeeding. The survival curve of women who perceived that their breast milk was inadequate were constantly below the survival curve of the other group *(log rank test, p < 0.05*) as illustrated in Fig. 3.

Using the multivariable Cox regression model, maternal education status, perceived inadequate breast milk, child feeding counseling during postnatal care, mode of delivery and wealth index were significantly associated with exclusive breastfeeding duration to six months. Women with a diploma level education and above are 2.9 times more likely to cease exclusive breastfeeding before six months child age as compared to illiterate women (AHR: 2.89, 95% CI:1.05, 7.97). Perceived inadequate breast milk among women increases the risk of early initiation of complementary feeding. Women who perceived inadequacy of their breast milk volume were 11 times more likely to cease exclusive breastfeeding earlier than their counterpart (AHR: 11.0, 95% CI: 6.70, 18.10). Postnatal breastfeeding counseling is significantly associated with exclusive breastfeeding duration. Women who are not counseled on exclusive breastfeeding during postnatal care are five times more likely to stop exclusive breastfeeding before six months child age as compared to women who received counseling (AHR: 5.05, 95% CI: 2.50, 10.23). Women who gave birth through cesarean section are more likely to terminate exclusive breastfeeding before six month as compared to women who had a spontaneous vaginal delivery (AHR: 3.8, 95% CI: 2.0, 7.2). Factors affecting exclusive breastfeeding duration are summarized in Table 3.

**Table 3:** Binary and Multivariable Cox regression model for predictors of exclusive breastfeeding duration among women who had 6–12 months old child in Gurage zone, South Ethiopia, 2016

Variable	Category	Exclusive breastfeeding duration status		CHR (95%, CI)	AHR (95%, CI)	*p- value*
		Event	Censored			
Maternal education status	Illiterate	51 (17.5)	240 (82.5)	1	1	
	Can read and write only	43 (24.2)	135 (75.8)	1.44 (0.96-2.15)	1.07 (0.56-2.07)	0.83
	Primary school first cycle (grade 1–4)	19 (19.2)	80 (80.8)	1.12 (0.66-1.89)	0.58 (0.22-1.54)	0.28
	Primary school second cycle (grade 5–8)	21 (21.4)	77 (78.6)	1.24 (0.75-2.06)	0.85 (0.36-1.98)	0.71
	Secondary school completed	19 (25.7)	55 (74.3)	1.59 (0.94-2.69)	1.24 (0.47-3.27)	0.66
	Diploma certified and above	17 (43.6)	22 (56.4)	3.13 (1.81-5.41)	2.89 (1.05-7.97)**	0.04
History of ANC	No	37 (52.9)	33 (47.1)	3.36 (2.33-4.83)	2.58 (0.99-6.65)	0.051
	Yes	133 (18.8)	575 (81.2)	1	1	
Place of delivery	Home	30 (42.3)	41 (57.7)	1	1	
	Health institutions	140 (19.8)	567 (80.2)	0.40 (0.27-0.66)	0.87 (0.10-7.64)	0.86
Mode of delivery	Cesarean section	14 (43.8)	18 (56.2)	2.6 (1.5-4.5)	3.8 (2.0 - 7.2)**	<0.0001
	Spontaneous vaginally	156 (20.9)	590 (79.1)	1	1	
Breastfeeding counseling during PNC	No	14 (60.9)	9 (39.1)	5.00 (2.84-8.80)	5.1 (2.5 – 10.23)**	<0.0001
	Yes	87 (14.5)	515 (85.5)	1	1	
Perceived adequacy of breast milk	No	57 (71.2)	23 (28.8)	5.8 (4.3 – 8.1)	11.0 (6.7-18.10)**	<0.0001
	Yes	113 (16.2)	585 (83.8)	1	1	
Wealth Index	Lowest quintile	40 (25.8)	115 (74.2)	1	1	
	Second quintile	28 (17.9)	128 (82.1)	0.64 (0.39-1.04)	0.48 (0.23-0.98)**	0.044
	Middle quintile	19 (12.2)	137 (87.8)	0.43 (0.25-0.74)	0.90 (0.40-2.04)	0.13
	Fourth quintile	33 (21.2)	123 (78.8)	0.77 (0.49-1.23)	1.10 (0.50-2.45)	0.77
	Fifth quintile	50 (32.2)	105 (67.7)	1.26 (0.83-1.90)	1.34 (0.64-2.80)	0.55

*CHR; Crude Hazard Ratio, *AHR: Adjusted Hazard Ratio, **Indicates statistically significant variables on a table

### Qualitative findings

The majority of points raised by focus group participants are summarized in the following themes. Traditional practices related to child care, maternal knowledge on proper child feeding, economic factors, maternal work load, maternal attitude towards exclusive breastfeeding previous experience of child feeding pattern, and influential others. However, there are responses which cannot be cascaded to neither of the aforementioned themes since list of themes is not exhaustive to represent all responses.

Significant number of participants mentioned the early cessation of exclusive breastfeeding in their community, and their own practices. There was active narration by participants about the early introduction of complementary feeding, as early as two months of child age and prelacteal feeding before the child start to suck breast milk. One participant explained “*In the first hours after birth I fed my child a solution made of sugar, water, salt, and tenadam, and when my breast started to pour milk I gave my breast milk”*. Another participant said “*I usually feed breast milk in the first six month of child age, but when I go far away from home I leave my child with boiled cow milk and care taker*.”

Perceived inadequacy of breast milk was echoed by majority of participants, and this was perceived as a cause for the child to cry and failure to thrive. One participant explains “*my child was continuously crying, shouting.....my neighbor told me that he was crying due to empty stomach (hungry), your breast milk is inadequate to satisfy the demand of the child, then I gave him soup, Zengada (*Sorghum like crop*) around three months of child age*.” Other participant added “*I gave additional food items around the age of three months because he was continuously crying due to hunger, my breast milk was not enough to satisfy his need.”.*

Maternal out door working status was pronounced by majority of respondents to terminate exclusive breastfeeding earlier than the appropriate age. *One participant said “I give breast milk as far as I am at home but when I leave home; the child feeds cow milk”.*

In addition to women who early cease exclusive breastfeeding; there are few women who extend exclusive breastfeeding beyond the recommended six months duration. One focus group participant reasoned this practice as *“my child was not comfortable to take other food items at six months of his age so that I gave him only breast milk till he is friendly to other food items”.*

## Discussion

The median duration of exclusive breastfeeding practice in this study is six months. One hundred seventy (21.9%) of women had stopped exclusive breastfeeding before six months child age. Maternal education of diploma and above, perceived inadequacy of breast milk and cesarean section mode of delivery are factors which significantly increase the risk of terminating exclusive breastfeeding practice, while receiving having postnatal counseling on child feeding, and belonging to house hold with a second wealth quintile significantly reduced the risk of exclusive breastfeeding cessation in the study area. Median duration of exclusive breastfeeding in this study is similar with studies done elsewhere in Ethiopia [10, 11]. However, this finding is higher than the median duration of exclusive breastfeeding in Kongo, Sri Lanka, India, China and Brazil [12–16]. This difference could be as a result of the difference in the study settings. In this study participants were mainly from rural and semi urban areas; while participants in the aforementioned studies were mainly urban residents who are more likely to be employed and to stay long time away from their home for a job.

Maternal education status is significantly associated with exclusive breastfeeding duration. Women with diploma and above are at higher risk to terminate exclusive breastfeeding earlier as compared to illiterate women. This may be explained as women with diploma and above are more likely to be employed and have limited time to stay with their children. This might force them to give supplementary food earlier. This finding is supported by a qualitative finding narrated by participants of focus group discussion. This finding is also supported by studies from Ethiopia and Philippines [11, 17]. In contrast to this finding, studies done in Nova Scotia and Sri Lanka indicated women with higher schooling status exclusively breastfeeding longer, as compared to the illiterate mothers [15, 18]. This scenario agrees with paradox of exclusive breastfeeding duration in relation to women education status in developing and developed countries. In developing countries, women with a higher education status are associated with shorter duration of exclusive breastfeeding, but in middle income and developed countries women with a lower education status are more likely to terminate exclusive breastfeeding earlier [17, 19].

Perceived inadequacy of breast milk is strong predictor of exclusive breastfeeding duration in this study. A significant proportion of women were anxious regarding the adequacy of their breast milk volume for their child. Consequently they provide supplementary food earlier than the appropriate age. A focus group participant illustrated this scenario “*My breast was dry (unable to give enough milk to meet the child's demand) my child continuously cry due to empty stomach (hungry) and I was forced to give him additional food around three months of age.”* This finding is supported by findings elsewhere in Ethiopia, Kenya, Indonesia and Brazil [14, 20–22]. In fact, if women have an adequate food intake and regularly breastfeed their children; the sucking action would increase breast milk production. However providing supplementary food might reduce the child’s tendency to suck, which further reduces milk production.

Women who gave birth by cesarean section are more likely to cease exclusive breastfeeding earlier as compared to women with spontaneous vaginal delivery. This finding is supported by other studies in Ethiopia, and Australia [23, 24]. This might be due to obstetric complications before and after cesarean section which may interfere with breastfeeding; furthermore the cesarean section wound pain and anesthesia may contribute to poor child nursing practices, including breastfeeding.

Infant feeding counseling during a postnatal visit is positively associated with the duration of exclusive breastfeeding. Moreover; one focus group discussant said *“health extension workers and health center workers informed us not to provide any food before six months of child age, other foods before six month is a disease for the child; and a child stomach is too soft to digest food other than breast milk*.” This is in line with findings elsewhere in Ethiopia [24, 25].

## Conclusion

An significant proportion of women cease exclusive breastfeeding before the recommended six months duration. Around one quarter of women had initiated complementary food before six months of child age. Maternal education of diploma and above, perceived inadequacy of breast milk, and a cesarean section mode of delivery are factors which significantly increase the risk of terminating exclusive breastfeeding. However having postnatal counseling on child feeding significantly reduces risk of exclusive breastfeeding cessation in the study area. Behavioral change communication should be enhanced to comply with the recommended exclusive breastfeeding duration and to address the misconceptions that hinder the practice of exclusive breastfeeding. Furthermore increasing the utilization of postnatal counseling on child feeding would have positive effect on the duration of exclusive breastfeeding.

## Abbreviations

AHR: Adjusted Hazard Ratio
AOR: Adjusted Odds Ratio
ANC: Antenatal care
CHR: Crude Hazard Ratio
CI: Confidence Interval
EBF: Exclusive Breastfeeding
EDHS: Ethiopian Demographic Health Survey
GBD: Global Burden of Disease
IYCF: Infant and Young Child Feeding
KM: Kilometer
PNC: Postnatal Care
WHO: World Health Organization
